# Integrated transcriptome and methylome analyses reveal the molecular regulation of drought stress in wild strawberry (*Fragaria nilgerrensis*)

**DOI:** 10.1186/s12870-022-04006-9

**Published:** 2022-12-28

**Authors:** Qiang Cao, Lin Huang, Jiamin Li, Peng Qu, Pang Tao, M. James C. Crabbe, Ticao Zhang, Qin Qiao

**Affiliations:** 1 School of Agriculture, Yunnan University, 650091 Kunming, China; 2 Horticultural Research Institute, Yunnan Academy of Agricultural Sciences, 650205 Kunming, China; 3 Wolfson College, Oxford University, Oxford, OX26UD UK; 4 Institute of Biomedical and Environmental Science and Technology, School of Life Sciences, University of Bedfordshire, Park Square, LU1 3JU Luton, UK; 5 School of Life Science, Shanxi University, 030006 Taiyuan, Shanxi China; 6 College of Chinese Material Medica, Yunnan University of Chinese Medicine, 650500 Kunming, China; 7 College of Horticulture and Landscape, Yunnan Agricultural University, 650201 Kunming, China

**Keywords:** *Fragaria nilgerrensis*, Strawberry, DNA methylation, RNA-Seq, Drought stress, WGCNA

## Abstract

**Background:**

*Fragaria nilgerrensis*, which is a diploid wild strawberry with excellent drought-resistance, would provide useful candidate genes for improving drought resistance of cultivated strawberry. So far, its molecular regulatory networks involved in drought stress are unclear. We therefore investigated the drought response regulatory networks of *F. nilgerrensis* based on the integrated analysis of DNA methylation, transcriptome and physiological traits during four time points under drought stress.

**Results:**

The most differentially expressed genes and the physiological changes were found at 8 days (T8) compared with 0 day (T0, control). Methylome analysis revealed slight dynamic changes in genome-wide mC levels under drought conditions, while the most hypomethylated and hypermethylated regions were identified at T4 and T8. Association analysis of the methylome and transcriptome revealed that unexpressed genes exhibited expected hypermethylation levels in mCHG and mCHH contexts, and highly expressed genes exhibited corresponding hypomethylation levels in the gene body, but mCG contexts showed the opposite trend. Then, 835 differentially methylated and expressed genes were identified and grouped into four clustering patterns to characterize their functions. The genes with either negative or positive correlation between methylation and gene expression were mainly associated with kinases, Reactive Oxygen Species (ROS) synthesis, scavenging, and the abscisic acid (ABA) signal pathway. Consistently, weighted gene co-expression network analysis (WGCNA) revealed Hub genes including *NCED*, *CYP707A2*, *PP2Cs* and others that play important roles in the ABA signaling pathway.

**Conclusion:**

*F. nilgerrensis* drought is dominated by ABA-dependent pathways, possibly accompanied by ABA-independent crosstalk. DNA methylation may affect gene expression, but their correlation was more subtle and multiple types of association exist. Maintaining the balance between ROS regeneration and scavenging is an important factor in drought resistance in *F. nilgerrensis*. These results deepen our understanding of drought resistance and its application in breeding in strawberry plants.

**Supplementary Information:**

The online version contains supplementary material available at 10.1186/s12870-022-04006-9.

## Background

Drought stress is a key environmental factor that adversely affects agricultural production [1, 2]. To tackle drought stress, plants have developed various adaptive mechanisms (drought escape, avoidance, tolerance and recovery) to enhance drought tolerance through evolution, and these adaptive mechanisms vary from the molecular up to the plant level [3]. More general responses of plants to drought stress include root system modulation, osmotic adjustment, antioxidant defense, solute accumulation, and abscisic acid (ABA) synthesis. At the early stage of water deficit, plants absorb as much water as possible from the soil by changing the root system architecture and close stomata to decrease water transpiration [4]. Then proline, glycinebetaine, and soluble sugar levels in plants are increased to enhance the capacity of osmotic adjustment to adapt to increased drought conditions [5–7]. Under severe drought stress, large amounts of reactive oxygen species (ROS) accumulate in plants, inducing intracellular oxidative stress and damage to cell membranes. Meanwhile, a variety of antioxidant enzymes such as superoxide dismutase (SOD), peroxidase (POD), and catalase (CAT) are produced to eliminate excess ROS and mitigate the effects of drought stress [8, 9]. In particular, various phytohormones regulate drought response by mutual interaction and regulation of transcription, in which ABA is the key hormone for plants to cope with drought stress [10, 11]. The accumulation of large amounts of endogenous ABA in plants activates the ABA signaling pathway, which regulates the expression of corresponding transcription factors, and downstream drought-related genes to promote stomatal closure and reduced transpiration [12, 13]. Although drought tolerance has been reportedly mediated by ABA-independent pathways in some plants, more and more drought response genes have been identified involving ABA-signaling pathways [14].

In addition, increased numbers of studies have shown that epigenetics plays an essential role in the response of plants to abiotic stresses [15]. DNA methylation, which is a primary mechanism for epigenetic variation, has been reported to contribute to adaptation to abiotic stresses in involving the heritable epigenetic markers, regulating gene expression and silencing transposons [16]. The pattern of DNA methylation changes in species-specific and tissue-specific ways under drought stress [17]. Methylomic and transcriptomic analysis of sea buckthorn under drought stress showed that drought-induced DNA methylation had regulatory effects on gene expression, including an ABA accumulation-related gene VSR6 [18]. Under drought stress, mung bean showed that genes with the lowest expression exhibited the highest CG, CHG and CHH methylation levels in the gene body and the highest expressed genes with moderate CG methylation [19]. These studies showed that plant gene transcription is closely associated with the changes in DNA methylation levels under drought stress.

Cultivated strawberry has a large leaf surface and a shallow root system, making it more sensitive to drought stress, which severely limits the yield and cultivation area of strawberry [20]. A previous study on response to drought stress of two strawberry cultivars showed that drought treatment enhances the contents of anthocyanin, proline, CAT, and SOD in strawberry leaves [21]. Furthermore, the drought tolerance of transgenic strawberry was enhanced by overexpressing the *RdreB1BI* gene, which could bind to the promoter region of *FvPIP2*, suggesting appropriate candidate genes would be helpful to improve drought resistance of strawberry by using a transgenic approach [22]. Wild strawberries are important resources holding a wealth of alleles related to traits of yield and stress resistance.

*Fragaria nilgerrensis* is a wild diploid strawberry with wide distribution in southwestern China [23]. *F. nilgerrensis* not only has great disease resistance and waterlogging tolerance, but also possesses white fruits with a special peach-like aroma, which can be used as an excellent source for improvement of strawberry varieties [24, 25]. More importantly, *F. nilgerrensis* has strong drought-resistance and can grow in arid areas [24]. This ability is not only contributed to by thick leaves, sturdy petioles, and dense yellow-brown sericeous on their whole plant, which could prevent water loss, but also determined by complex regulatory gene networks [24, 26]. Thus, it is worthwhile to study the drought-resistant mechanisms of *F. nilgerrensis* with integrated strategies to highlight the genes that could potentially mitigate the stress, and which could be useful for improving drought resistance of the cultivated strawberry. Following omics technology improvements, integrated strategies involving traditional physiological and multi-omics approaches can be utilized to decode the molecular regulatory networks involved in drought stress. In this research, we investigated gene expression profiles and whole genome DNA methylation of *F. nilgerrensis* at different time points of drought stress treatment. Through the integration of transcriptome and methylome, this work not only improves our understanding of the molecular mechanisms of strawberry drought resistance, but also provides a foundation for further investigating and facilitating the breeding of drought resistant strawberry plants.

## Results and discussion

### Physiological traits of *F. nilgerrensis* under drought stress

**Fig. 1:**
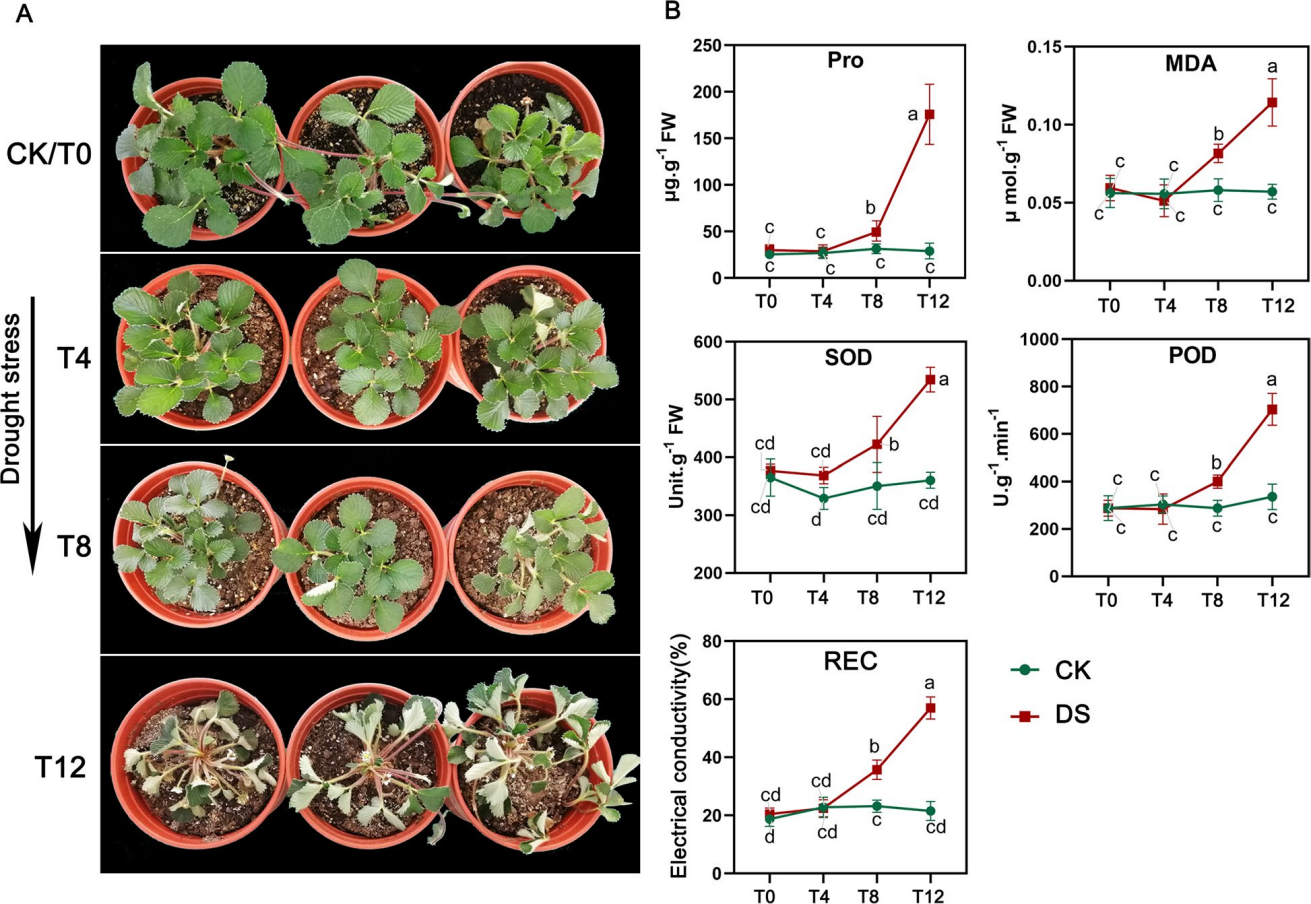
Drought stress experimental materials. (**A**) Morphological changes of *F. nilgerrensis* in response to drought; (**B**) Determination of five physiological traits at four time points of drought. Significant differences from five replicates are indicated by different letters (*P* value < 0.05, ANOVA)

Harmful ROS content usually increases dramatically under drought stress and attacks membrane lipids, accompanied by peroxidative damage to DNA, proteins, and membrane lipids [27]. To resist oxidative damage, plants have gradually evolved many protective scavenging or antioxidant defense mechanisms, such as SOD and POD antioxidant enzymes [28]. In this study, the contents of malondialdehyde (MDA) and relative electrical conductivity (REC), which are indicators of oxidative damage [29], were significantly increased at T8 (*P* < 0.05) and continued to increase sharply at T12 (*P* < 0.01) (Fig. 1 A, B), indicating that *F. nilgerrensis* was probably suffering from severe oxidative damage from T8. Correspondingly, the activities of POD and SOD also displayed an increasing tread from T8 to T12, which may be in counteracting the damage caused by ROS. Besides these enzymes, we also found a significant rise in proline (Pro) content from T8 to T12 in *F. nilgerrensis*, which can modulate intracellular and extracellular osmotic potential to improve plant water retention [30]. The smallest increase of all the five parameters (Pro, MDA, SOD, POD, REC) were detected at T4. These drought-inducible physiological changes indicated that multiple physiological processes, which were regulated by molecular alterations, were initiated near or at T8.

### Changes of differentially expressed genes (DEGs) under drought stress

To explore the drought resistance mechanism of *F. nilgerrensis*, we sequenced the transcriptome and whole genome bisulfite sequencing of leaves at four time points (T0, T4, T8, and T12). Among them, transcriptome sequencing was completed in 11 samples with an average of 42.5 million clean reads (~ 6.4Gb) per sample, which were subsequently mapped to the *F. nilgerrensis* genome. The mapping rate was as high as about 95%, and the unique mapping rate was greater than 92% on average (Supplementary Table S1). The Pearson correlation coefficients (R^2^) among replicates mostly exceeded 0.9, indicating the high reproducibility between replicates of each time point (Supplementary Fig. S1A). Although only 2 replicates in T0 for RNA-seq analysis were used due to original data damage, Pearson correlation coefficients (R^2^) and Quantile-Quantile Plot statistical model tests of these two replicates showed that both data were consistent and correlated well (Supplementary Fig. S1A & B).

**Fig. 2:**
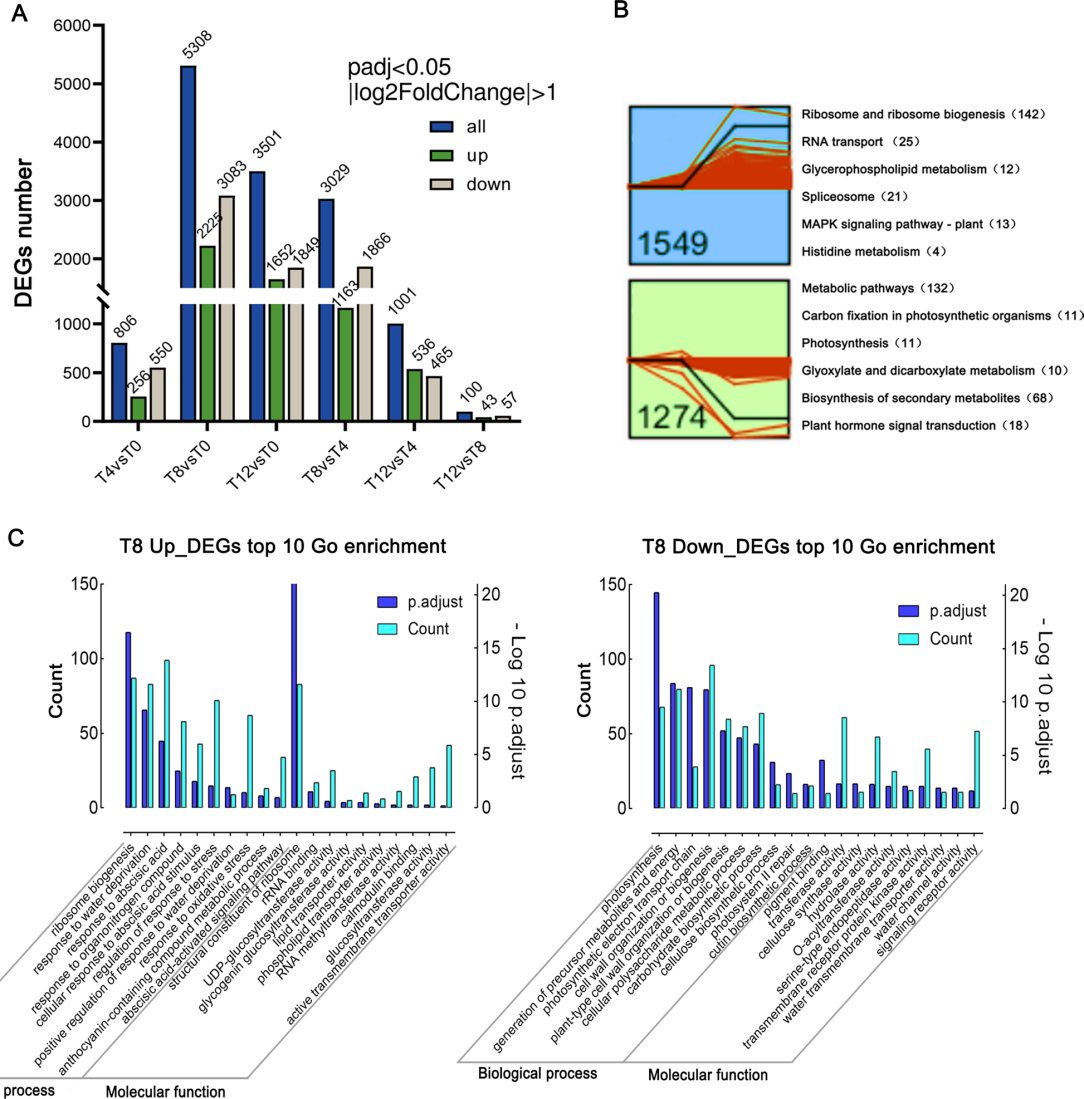
Differentially expressed genes (DEGs) detection and functional annotation. (**A**) The number of up- and down-regulated DEGs at each time point; (**B**) Two significant clusters were obtained at day 8 (T8) of drought. KEGG enrichment of two clusters with number of unigenes enriched in each pathway are also shown; (**C**) The top 10 GO enrichment analysis of DEGs between T8 and T0

According to the results of transcriptome sequencing, the number of differentially expressed genes (DEGs) was increased with the aggravation of drought stress. Compared with T0, the most DEGs (5,308) were detected at T8, of which the numbers of significantly up-regulated and down-regulated genes were 2,225 and 3,083, respectively (Fig. 2A). Notably, the lowest number of DEGs was found between T12 vs. T8 (100 DEGs), suggesting the gene expression patterns of T8 and T12 were similar. Consistently, a Venn plot showed that T8 and T12 shared most DEGs (2,593) (Supplementary Fig. S1C) as well as the hierarchical cluster analysis of DEGs, which showed that T8 and T12 were clustered together (Supplementary Fig. S1D). These results further indicated that dramatic molecular and metabolic alterations of *F. nilgerrensis* occurred at T8. Then we searched common temporal expression patterns using the Short Time-series Expression Miner (STEM) program [31] and found 11 significant profiles (Supplementary Fig. S1E), among which two broad profiles exhibited up (1,549 genes) or down (1,274 genes) regulation at T8 (Fig. 2B). Notably, the Kyoto Encyclopedia of Genes and Genomes (KEGG) enrichment analysis revealed that up-regulated genes were enriched in the glycerophospholipid metabolic pathway, MAPK signaling and ribosome biosynthesis, while down-regulated genes were enriched in carbon fixation and photosynthesis (Fig. 2B). Previous studies have shown that the MAPK cascade is a key signal mechanism in response to various external stimuli, including drought stress [32]. The Glycerophospholipid metabolic pathway has also been reported in drought tolerance studies on the tolerant diploid common wheat ancestor *Aegilops tauschii* [33]. In contrast, stomatal closure under water-deficient conditions allows plants to minimize water loss by transpiration, and with the inaccessibility of CO_2_, the rate of photosynthesis decreased [34]. Thus, these genes  may play crucial roles in response to drought in *F. nilgerrensis.*

Consistently, if we focused on DEGs at T8 vs. T0, a larger number of pathways were enriched in drought resistance according to GO (Gene Ontology) enrichment analysis. The up-regulated genes were mainly enriched in response to water deprivation, oxidative stress, abscisic acid-activated signaling pathway, UDP-glucosyltransferase activity, calmodulin binding, and active transmembrane transporter activity (Fig. 2C). The down-regulated genes were mainly enriched in photosynthesis, cell wall organization or biogenesis, transmembrane receptor protein kinase activity and water channel activity. The main enrichment pathways involved in DEGs at the T8 time point roughly delineated the gene involvement process of *F. nilgerrensis* drought resistance.

### Methylation landscape of *F. nilgerrensis* under drought stress

To explore the regulatory mechanisms of methylation levels after drought, we simultaneously performed whole genome bisulfite sequencing (WGBS) analysis. A total of 126Gb clean data were produced for 12 samples, with an average of ~ 10.5Gb per sample. The sequencing depth was more than 30 × (305.9 Mb for the genome), and the unique mapping rate ranged from 67.75 to 73.13%. The lowest Q20 and Q30 were 96.78% and 90.18%, and the bisulfite conversion rate ranged from 99.499 to 99.782%, indicating that the DNA methylation information on reads was highly reliable (Supplementary Table S2).

**Fig. 3:**
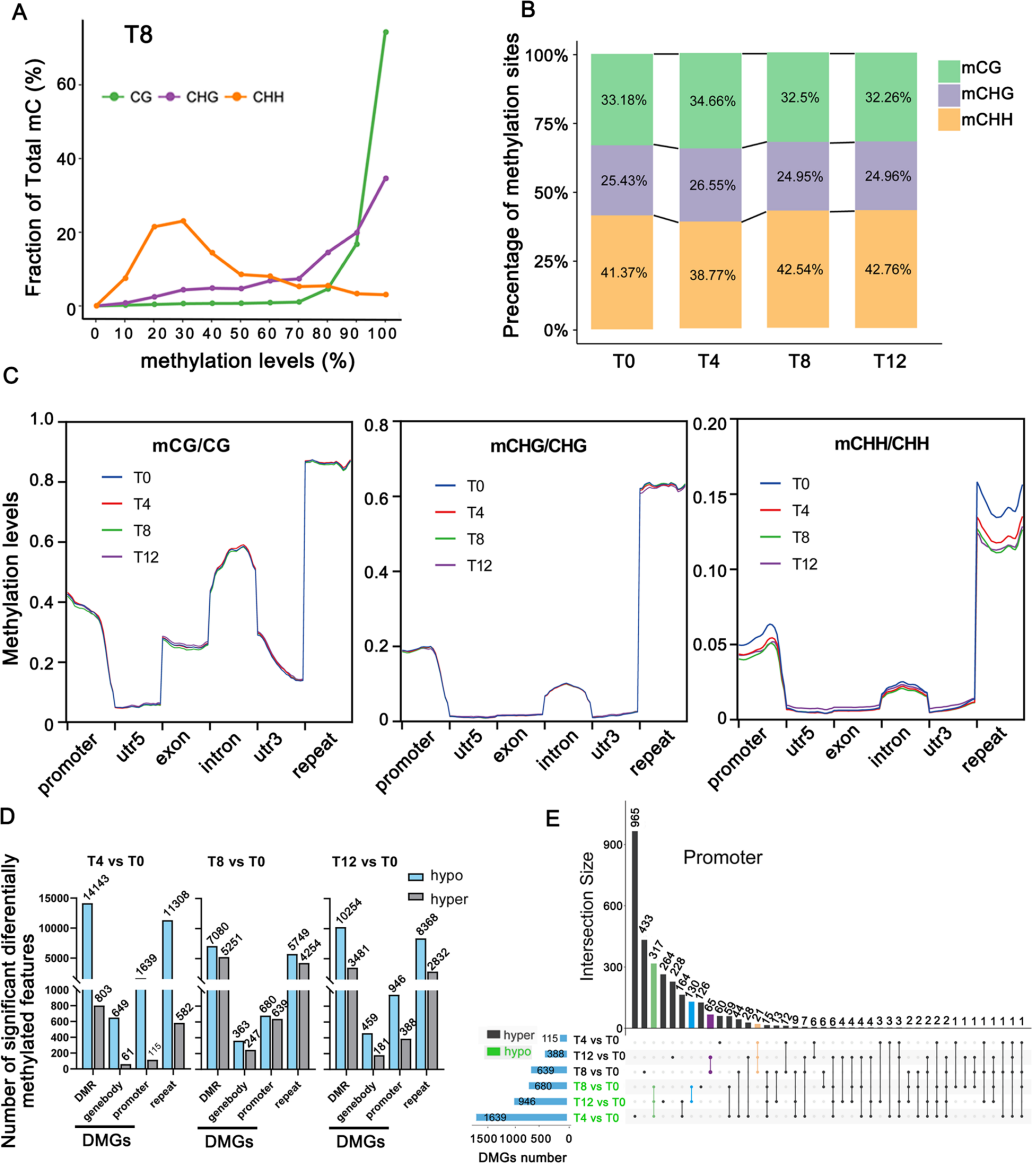
The dynamics of methylation levels at four time points of drought. (**A**) The distribution of methylation levels in different sequence contexts at T8. The x axis represents the methylation level, and the y axis represents the fraction of total methylated cytosine sites; (**B**) Relative proportions of mCs in CG, CHG and CHH contexts at different time points of drought; (**C**) Dynamic changes in DNA methylation including gene components and repeats; (**D**) The number of differentially methylated regions (DMRs) and their corresponding genes (DMGs) were counted for each comparison time point; (**E**) Upset Venn plot of promoter methylation-related genes in each comparison  group

The average methylation level across the genome is methylated mC divided by the sum of mC and unmethylated umC at certain cytosine sites. Among three sequence contexts of *F. nilgerrensis*, the CG context exhibited highest methylation level, followed by the CHG and CHH contexts, with an average of 47.69%, 30.87% and 10.56%, respectively (Fig. 3 A, Supplementary Table S3). In addition, we also calculated the percentage of three contexts (mCG, mCHG and mCHH) in the total mC sites and found they exhibited dynamic changes at various time points, of which mCHH not only accounted for the highest percentage but also showed the biggest changes (Fig. 3B) (Supplementary Table S3). This was consistent with previous findings in *Morus alba* and *Gossypium hirsutum* that CHH methylation level is dynamic with environments and that CHH methylation may be closely correlated with drought stress [35, 36]. Consistently, DNA methylation levels of gene components and repeats also showed significant changes in CHH methylation during drought stress in *F. nilgerrensis*, especially in promoters and repeat sequences (Fig. 3C).

The sum numbers of differentially methylated regions (DMRs) between each time point and the control (T0) were also determined and most hypo- and hypermethylated DMRs were detected at T4 and T8 respectively (Fig. 3D). These DMRs mainly occur in the CHH context, and about 80% of them were in repeat elements (Supplementary Fig. S2A). Accordingly, the methylation levels for each element in different genomic regions showed that repeat elements showed the highest methylation levels in all three contexts and all the time points, followed by promoters and introns (Fig. 3C). As the methylation at promoter and gene body could have different effects on gene expression, we have classified differentially methylated genes (DMGs) as promoter DMGs and gene body DMGs. The results showed that promoter DMGs were more than gene body DMGs in *F. nilgerrenis* at all the time points of drought stress (Fig. 3D). Among the promoter DMGs, 317 hypo- and 21 hyper-methylated genes were shared across all the time points respectively (Fig. 3E), suggesting the methylation level of these genes changed dynamically and may be involved in the regulation of expression genes in response to drought stress directly or indirectly. KEGG enrichment showed that these genes were mainly involved in plant hormone signal transduction, the MAPK signaling pathway and the ubiquitin mediated proteolysis pathways (Supplementary Fig. S2B). These results were roughly consistent with KEGG enrichment of DEGs.

### The relationship between methylation level and gene expression in response to drought stress

**Fig. 4:**
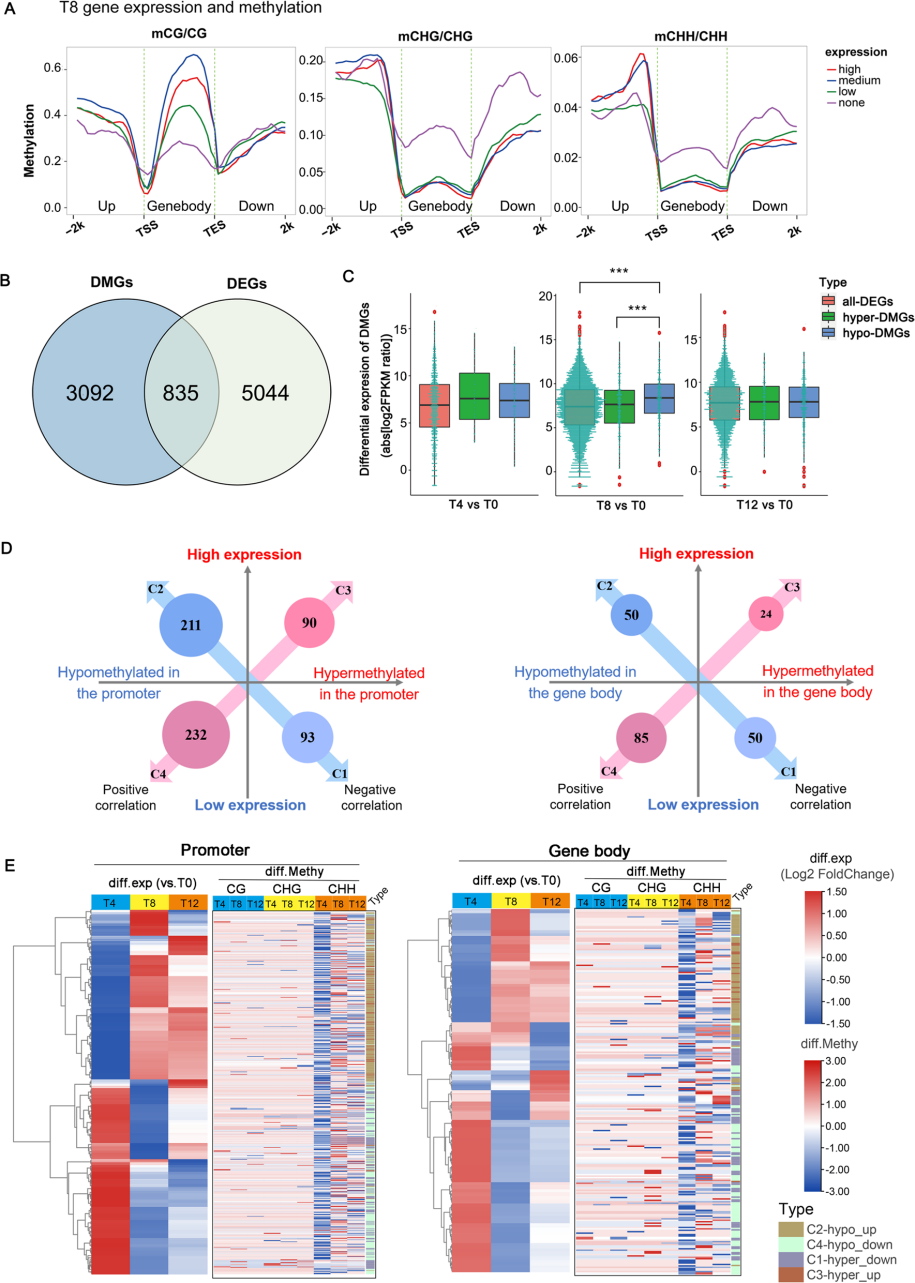
Integrative analysis of methylome and transcriptome revealed expression regulation of drought response genes. (**A**) Methylation levels of genomic fractions (gene bodies and their upstream and downstream 2 kb regions) of different expression classes: low to high expression levels correspond to the lower, middle, and upper quartiles of FPKM, with FPKM < 1 indicating no expression (none); (**B**) The shared 835 genes were subjected to methylome-transcriptome association analysis; (**C**) Box plots of gene expression levels of all DEGs, hypermethylated and hypomethylation-DGEs. The turquoise dotplot in the middle represents gene numbers and corresponding expression levels. *** *P*.adj < 0.001 (ANOVA, Tukey_HSD); (**D**, **E**) Identifies the association of promoter and gene body methylation with the expression of 835 genes. C1: Hypermethylation and low expression; C2: Hypomethylation and high expression; C3: Hypermethylation and high expression; C4: Hypomethylation and low expression. Detailed gene lists are shown in supplementary data Table 4

Dynamic changes in DNA methylation are crucial in regulating gene expression of plants under abiotic stresses [37, 38]. Taking T8 (containing most differentially methylated expressed genes, DMEGs) for example, we explored the association between DNA methylation and expression in different genomic regions and different contexts. The genes detected in the transcriptome were divided into four categories according to their expression levels: no expression (FPKM [fragments per kilobase of transcript sequence per millions base pairs sequenced] < 1), low expression, medium expression and high expression level, of which the last  three corresponded to the lower, middle and upper quartiles of FPKM. We found that the unexpressed genes exhibited expectable high methylation levels and highly expressed genes showed low methylation levels correspondingly in the gene body except for the CG methylation, which exhibited an opposite trend (Fig. 4A). Consistent with this, CG gene body methylation has been reported to be positively correlated with gene expression in angiosperms, while CHG and CHH gene body methylation is associated with reduced gene expression [39, 40]. Moreover, GC gene body methylation was found in moderate and constitutively expressed housekeeping genes, although it is unclear whether it has a function [41, 42]. Conversely, genes with medium to high expression levels in all three contexts showed high methylation levels 2 kb upstream of the transcription start site (TSS), indicating that DNA methylation was positively correlated with gene expression in the promoter region (Fig. 4A). It is notable that all three contexts exhibited the lowest methylation levels near TSS and transcription termination sites (TES). This was consistent with previous reports that the lack of methylation around transcription initiation and termination sites appears to be important for gene expression regulation [43, 44]. Further, we also divided the methylation levels of these genes into five groups by quintile, with the first group being the lowest and the fifth group the highest (Supplementary Fig. S3A). This reverse verification and analysis gave the same results as above.

To further characterize the relationship between DNA methylation and gene expression, we merged DMGs and DEGs of all the time points and identified 835 DMEGs (Fig. 4B, Supplementary Table S4). Analysis of the expression levels of DMEGs at different time points showed that the average expression level of hypomethylated DMGs at the T8 time point was significantly higher than that of hypermethylated DMGs and all DEGs (*P* < 0.001, without distinguishing specific methylated regions), indicating that hypomethylation contributed to the increase in gene expression levels at T8 (Fig. 4C). This was consistent with the fact that most recognized hypomethylation usually promotes gene expression [37, 45]. On the contrary, DMEGs at T4 and T12 showed a slight increase in gene expression after hypermethylation, but it was not significant. In order to further explore the correlation between methylation and expression, these genes were systematically classified into four different clusters (C1, C2, C3 and C4, respectively) based on hyper- and hypo-methylation in promoter and gene body as well as gene expression patterns (Fig. 4D). Among promoter and gene body DMEGs, both positive and negative correlations were found in methylation and gene expression. In promoter DMEGs, the genes of C1 and C2 showed typical methylation regulation patterns, i.e., hypermethylation represses the gene expression while hypomethylation promotes expression; in contrast, the genes of C3 and C4 showed positive correlation in methylation and gene expression (Fig. 4D). Cluster analysis of expression and methylation of DMEGs revealed that CHH methylation of these DMEGs changed dramatically under drought stress (Fig. 4E). It is noteworthy that expression of most genes in C2 was upregulated at T8 and T12, while that of C4 was downregulated at T8 and T12, with either promoter or gene body methylation (Fig. 4E). The functions of these gene groups showed that many genes in C2 (Hypomethylation and high expression) and C3 (Hypermethylation and high expression) were related to hormone signaling, kinases, transcription factors, detydrin, and detoxificants, while C1 (Hypermethylation and low expression) and C4 (Hypomethylation and low expression) contained a large number of photosynthesis-related genes (Supplementary Fig. S3B). It suggested that hypomethylation may play a major role in response to drought stress in *F. nilgerrensis*. Meanwhile, it also indicated that methylation has multiple types of associations with gene expression, which are subtle and not simply linearly correlated. In rice, promoter DNA methylation has been reported to be associated with transcriptional repression, while gene body methylation usually upregulates gene expression [45]. In contrast, positive associations of DNA methylation to gene expression were found in both promoter and gene body in apple [46]. In phosphate starvation studies, gene transcriptional changes occurred even prior to differential methylation [47]. This may imply a deep relationship between DNA methylation and gene expression and is not just a case of maintaining genome stability and suppressing gene expression as in previous reports [36, 46].

### Characterization of key genes for drought response in *F. nilgerrensis*

**Fig. 5:**
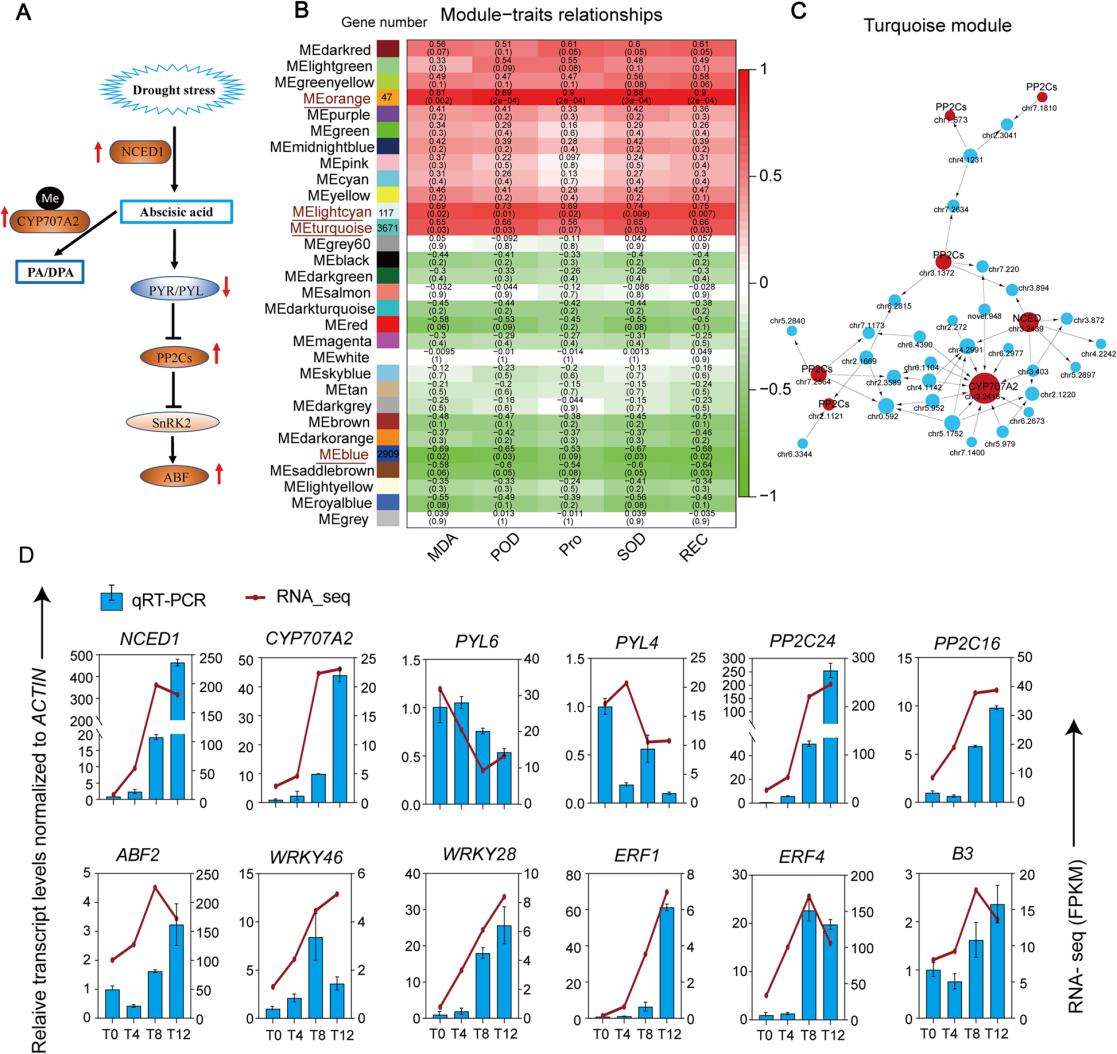
Weighted gene co-expression network analysis (WGCNA) reveals drought resistance modules and qRT-PCR validation of key drought resistance genes. (**A**) A model of ABA signaling regulation under drought in *F. nilgerrensis.* The upper and lower red arrows indicate up- and down-regulation of genes, respectively, and “Me” indicates differential methylation; PA/DPA: Phase acid and dihydro phase acid; (**B**) Module-trait correlation. Each column corresponds to a module indicated by a different color, each row corresponds to a drought physiological characteristic, and the intersecting cell numbers indicate correlation and *P* value; (**C**) Correlation network of highly correlated turquoise modules. The core components genes of ABA signalling are characterized by “red”, and the weight is characterized by the size of the node, which reflects the number of genes related to it; (**D**) The expression level of response genes under drought stress was validated by real-time quantitative PCR. Bars represented means ± SD from three biological replicates

**Fig. 6:**
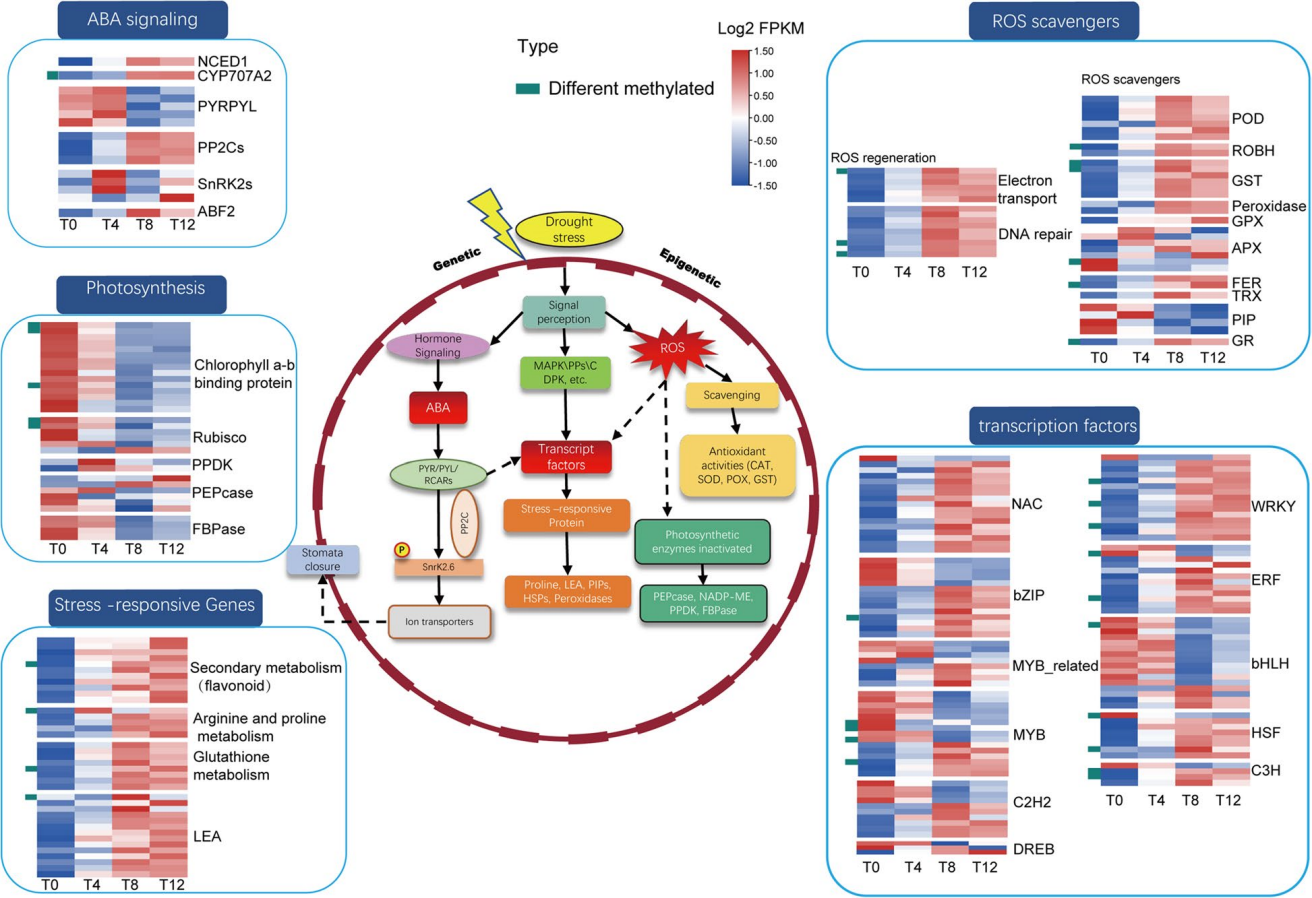
The drought response regulatory networks of *F. nilgerrensis*. The green bar on the left of the heatmap indicates differential methylation occurred. *RuBisco*: Ribulose-1,5-bisphosphate carboxylase, *PPDK*: Pyruvate, phosphate dikinase, *PEPcase*: Phosphoenolpyruvate carboxykinase, *LEA*: Late embriogenesis abundant protein, *FBPase*: Fructose-1,6-bisphosphatase, *POD*: Peroxide Enzyme, *ROBH*: Respiratory burst oxidase homolog, *GST*: Glutathione transferase, *GPX*: Glutathione peroxidase, *FER*: Ferritin, *TRX*: Thioredoxin, *PIP*: Aquaporins, *APX*: Ascorbate peroxidase, *GR*: Glutathione reductase

The GO enrichment analysis of DEGs indicated that the ABA signaling pathway was significantly enriched in the drought response of *F. nilgerrensis* (Fig. 2C). ABA signaling is known to respond to stress in drought by regulating stomatal closure and gene expression [48, 49]. Notably, the expression of gene *NCED1* encoding 9-cis-epoxycarotenoid dioxygenase (an important enzyme in the ABA biosynthetic pathway) and gene *CYP707A2* encoding ABA 8’-hydroxylase (a key enzyme in the oxidative catabolism of ABA) were significantly upregulated by more than 10-fold (Fig. 5 A), in which *CYP707A2* was found to be accompanied by hypomethylation in the promoter region throughout drought stress. Significant upregulated expression of *NCED1* and *CYP707A2* and an increased ABA content was also detected in cultivated strawberries under drought stress (*F. × ananassa*) [50]. This suggests that drought stress on *F. nilgerrensis* promotes ABA biosynthesis and activates ABA-dependent signaling pathways. It has been found that under stress conditions, ABA levels increased and the PYRABACTIN RESISTANCE/PYR-LIKE/REGULATORY COMPONENTS OF ABA RECEPTORS (PYR/PYL/RCAR) bound to ABA to interact with and inhibit a downstream target, the clade A type 2 C protein phosphatase (PP2Cs), thereby releasing the SNF1-related protein kinase 2 (SnRK2) [51]. The released SnRK2 (especially encoded by subclasses III and II of SnRK2) eventually leads to the phosphorylation of the downstream ABA-dependent signaling gene ABF/AREB to activate the stress response [52]. Our results showed that expression of five ABA receptor genes (*PYL6*, *PYR1*, *PYL8*, and two *PYL2s*) genes were down-regulated, while expression of PP2Cs, genes in subclass II of SnRK2 and *ABF2* were significantly up-regulated in *F. nilgerrensis* under drought stress (Figs. 5A and 6, Supplementary Fig. S4). In contrast to the common expectation that ABA reduces the expression of PYR/PYLs receptors and induces the expression of PP2Cs, no common trend between ABA content and PYR/PYLs expression has been detected in some species, which appears to be the mechanism for reducing persistent ABA damage [53]. This pattern was also observed in *Arabidopsis* that the expression levels of PYR/PYLs were down-regulated, while PP2Cs and ABFs were up-regulated under stress [54]. Consistently, most PYLs were down-regulated except for *ZmPYL4*, *ZmPYL7* and *ZmPYL8* in polyethylene glycol (PEG)-treated maize leaves, and PP2Cs and SnRK2s were either up-regulated or unchanged [55]. The lack of differential expression of SnRK2 subclass III genes in *F. nilgerrensis* may be caused by a missed right time point, and the activation of the downstream target gene *ABF2* also supported this speculation. It was reported that subclass III genes of SnRK2 were rapidly activated within minutes after exogenous ABA administration in *Arabidopsis* [56].

We performed a weighted gene co-expression network analysis (WGCNA) on the RNA-seq data, using the hierarchical clustering method, to identify co-expression modules and link these to physiological traits. A total of 29 valid modules (grey is an invalid module) were obtained (Supplementary Fig. S5A, B). We found that the orange, lightcyan and turquoise modules were significantly positively correlated with the five physiological traits, with an average correlation of 0.76 (*P* < 0.05) (Fig. 5B). In contrast, the blue module showed a significantly negative correlation with these physiological traits. The KEGG enrichment analysis showed that the positively correlated three modules were mainly enriched in ubiquitin mediated proteolysis, amino acid biosynthesis and the MAPK signaling pathway, while the negatively correlated blue module was mainly enriched in photosynthesis, phytohormone signal transduction and biosynthesis of secondary metabolites (Supplementary Fig. S5B).

Genes with high K_ME_ (eigengene connectivity) values in the co-expression network generated by WGCNA are possible hub genes, which are significantly correlated with physiological traits. More importantly, a large number of important genes in the ABA signaling pathway were also identified in the turquoise color module, including *NCED1*, *CYP707A2*, and *PP2Cs*, which indicated that the ABA pathway plays an important role in regulating physiological response in *F. nilgerrensis* under drought stress (Fig. 5C, D). It is notable that the *FaNCED* gene was reported to be expressed only in roots, but not in leaves in cultivated strawberry [57], while in our study we detected a high expression of *NCED* in leaves of *F. nilgerrensis* under drought. Although the root-derived ABA theory has been widely accepted, recent study has shown that leaves are considered to be the initial source of ABA biosynthesis during water stress due to the presence of large amounts of endogenous carotenoid precursors required for ABA biosynthesis [58]. Besides, the hub genes of the orange module, such as *WRKY28*, Zinc finger protein 7 (*ZFP7*), and Peroxiredoxin-2B (*PRXIIB*) have also been shown to be highly related to drought resistance (Supplementary Fig. S5C). Among them, *ZEP7* was reported to regulate ABA signaling in *Arabidopsis*, and *WRKY28*, which co-expressed with *AtbHLH17*, were known to be up-regulated under drought and oxidative stress, improving resistance to abiotic stress in *Arabidopsis* [59]. Meanwhile, *PRXIIBs* have been reported to play a significant role in cyto-protection against oxidative stress by eliminating peroxides and acting as sensors of hydrogen peroxide-mediated signaling events [60].

In addition, drought usually prevents the entry of CO_2_ into leaves and finally results in a decrease of photosynthetic rate [61]. Consistent with this point, a large number of key photosynthesis-related genes showed significantly down regulated expression and differential methylation in our results, such as the genes encoding fructose 1,6-bisphosphatase (*FBPase*), chlorophyll-binding protein (*CBP*) and ribulose-1,5-bisphosphate carboxylase (*RuBisco*) (Fig. 6), which are important regulators of photosynthesis [62]. Furthermore, drought-induced stomatal closure, reduced carbon dioxide uptake, reduced photosynthetic rate, and changes in chloroplast photochemical reactions could also cause overproduction of ROS [63]. Consistently, we observed that expression of respiratory burst oxidase homologs (*RBOH*) was significantly upregulated with or without methylation alteration (Fig. 6), which were reported to be the key genes for ROS synthesis and play a crucial role in their responses to biotic and abiotic stresses [64, 65]. Excessive ROS would cause oxidative stress to impair DNA, proteins and lipids, resulting in cell dysfunction and death [66]. Thus, ROS levels under stress conditions are associated with ROS production and ROS clearance maintenance, which also represents the ability of the stress response [67]. Our results showed that in response to possible ROS damage, ROS scavenging systems were activated and expression of genes such as *POD*, glutathione transferase (*GST*), glutathione reductase (*GR*) and glutathione peroxidase (*GPX*) were significantly increased, regulated by different methylation (Fig. 6). It indicated that a rapid and effective antioxidant defense system involved in ROS detoxification in *F. nilgerrensis* under drought stress may provide strong resistance.

Transcription factors (TFs) are inevitably activated in response to transduction of stress signals and subsequently trigger the expression of a large number of stress-responsive genes [68, 69]. To illustrate the changes in expression and methylation of TFs under drought stress, 1,285 TFs in *F. nilgerrensis* genome were identified according to the transcription factor database PlantTFDB (http://planttfdb.gao-lab.org), among which 189 TFs were differentially expressed under drought stress (Supplementary Table S5). These differentially expressed TFs were from 34 gene families, including NAC (19 members), WRKY (15 members), MYB (23 members), bZIP (14 members), ERF (13 members) and DREB (7 members). The heat map showed a large number of TFs exhibited significantly increased expression under drought stress, including NAC, WRKY, HSF and ERF, and that some of them were accompanied by differential methylation (Fig. 6). Interestingly, we identified a significant upregulation of dehydration response element binding (*DREB*), which is a key TF in the regulation of the ABA-independent pathway. This may imply that there are both ABA-dependent and -independent signals in *F. nilgerrensis* for drought response.

Among these differentially expressed TFs, 25 TFs also showed methylation changes in the promoter region, including the heat stress transcription factor A-6b, the AP2/ERF family transcription factor *ERF4*, and the possible WRKY transcription factor 46 (Supplementary Table S6). Notably, we found that *ERF1*, *WRKY46*, *B3* domain-containing and *Dof3.6* showed continuously increased expression and hypomethylation across all the time points. Among them, *ERF1* was reported to be able to integrate stress-specific gene regulation of multiple hormonal signals to play an active role in drought and heat stress tolerance in *Arabidopsis* [70]. Previous studies have shown that *WRKY64* is involved in stress osmotic and stomatal regulation [71]. Besides, *B3* domain-containing [72] and *Dof* [73] also have been reported to be involved in drought stress. These results suggest that these TFs should play a central role in drought stress tolerance in *F. nilgerrensis*. Finally, we randomly selected the above 12 genes (*NCED1*, *CYP707A2*, two each of *PYLs* and *PP2Cs*, *ABF2*, *WRKY46*, *WRKY28*, *ERF1*, *ERF4* and *B3*) for qRT-PCR verification, and obtained results consistent with the transcriptome trend (Fig. 5D).

## Conclusion

*F. nilgerrensis* is an important wild source for improvement of the drought resistance of cultivated strawberry plants. In this study, the drought response regulatory networks of *F. nilgerrensis* were revealed based on the integrated analysis of DNA methylation, transcriptome and physiological traits. Our study demonstrated that both ABA-dependent and independent signaling pathways play roles in the drought response in *F. nilgerrensis*. Besides, the ability of osmotic adjustment and the maintenance of a balance between ROS regeneration and scavenging was also crucial to prevent metabolic dysfunction and greatly determined overall drought tolerance of *F. nilgerrensis*. In addition, the correlation between DNA methylation and gene expression was more subtle, showing both positive and negative correlations regardless in both promoters and gene bodies. That suggests multiple types of association exist between DNA methylation and gene expression. In summary, our study offers a model for a comprehensive exploration of the drought resistance mechanisms of plants, and also provides a reference for manipulation in breeding and crop management.

## Materials and methods

### Plant material and drought stress treatment

Plants of *F. nilgerrensis* were collected from Liangwang Mountain, Yuxi, Yunnan Province, China (102°54.910′ E, 24°45.474′ N), and grown in the greenhouse of the College of Agriculture, Yunnan University, ensuring that the experimental material was from the same clone (The plant specimens are kept in the Herbarium of Yunnan University, and the identification was undertaken by Pro. Qin Qiao [74]). No specific permissions and licenses were required for the collection of the material. Drought experiments were conducted in a conservatory at 20–26℃ and 55-68% relative humidity. These plants were divided into control and treatment groups and treated as follows: (i) control group: plants were watered every 2d; (ii) drought treatment group: plants were not watered from 0d to 12d. Leaves of *F. nilgerrensis* were collected from four time points with three biological replicates during continuous drought stress: 0d (T0, CK), 4d (T4), 8d (T8) and 12d (T12) (Fig. 1 A). The leaves were sampled between 9 and 10 a.m. at each time point under drought stress. In the meantime, the materials utilized for DNA methylation, transcriptome analysis and qRT-PCR were obtained from three biological replicates at four time points, which were frozen in liquid nitrogen promptly and then stored at -80℃.

### Physiological traits measurement

Each physiological index was examined with 100 mg fresh leaf samples respectively. Proline content was analyzed following the acid ninhydrin procedure modified by Bates, et al. [75]. Proline content was calculated as follows: (µg proline/ml × ml toluene) / (0.1 g × ml sample) = µg proline/g of fresh weight material. The content of malondialdehyde (MDA) was determined as described by Zhao, et al. [76]. The activity of POD was measured using the guaiacol oxidation method [77]. The total SOD activity was assayed according to the method described by Giannopolitis and Ries [78]. The measurement of REC was referred to the method described by Zhou, et al. [79]. One-way ANOVA of physiological parameters was based on SPSS 26.0.

### Whole genome Bisulfite sequencing (WGBS)

Total genomic DNA was extracted using the Hi-DNAsecure Plant Kit (Qiagen GmbH, Hilden, Germany). A total amount of 5.2 µg genomic DNA spiked with 26 ng lambda DNA was fragmented by sonication to 200-300 bp with Covaris S220, and then treated twice with sodium bisulfite using the EZ DNA Methylation-Gold Kit (Zymo Research). Meanwhile, PCR amplification of single-stranded DNA fragments was performed using KAPA HiFi Hot Start Uracil + Kit-mix (2X) to obtain the BS seq library. Subsequently, BS_seq library concentrations were quantified using a Qubit® 2.0 fluorometer (Life Technologies, CA, USA) and insert sizes were determined on a Bioanalyzer 2100 system (Agilent). The prepared BS_seq libraries were then sequenced on the Illumina Hiseq2500/4000 or Novaseq platforms to generate 125 bp of paired-end sequences.

Quality control of the raw data obtained from sequencing was performed using FastQC (fastqc_v0.11.5). The clean data were then mapped to the *F. nilgerrensis* genome [74] using Bismark software (0.16.3) for methylation site detection [80]. The *F. nilgerrensis* genome was transformed into bisulfite transformed versions (C-to-T and G-to-A transformations), indexed using bowtie2 [81] constructs, and subsequently compared to BS_seq reads against each other to determine the unique best alignment. Differential methylation regions (DMRs) were identified using the biological software package DSS (smoothing.span = 200, delta = 0, p.threshold = 1e-05, minlen = 50, minCG = 3, dis.merge = 100, pct.sig = 0.5) [82]. GO enrichment and KEGG enrichment analysis of DMGs were performed using clusterProfiler R package [83] and KOBAS software [84], respectively.

### Transcriptome sequencing

Four time points of leaf RNA extraction were performed using the Tenagen polysaccharide polyphenol kit (QIAGEN, Germany), according to the manufacturer’s instructions. RNA was quality-checked using an Agilent 2100 bioanalyzer and qualified RNA was used for library construction, referring to the instructions of the kit NEBNext® Ultra™ RNA Library Prep Kit for Illumina® for library construction [85]. To ensure the quality of the RNA_seq library, cDNA fragments of 370–420 bp in length were selected using AMPure XP beads (Beckman Coulter, Beverly, USA) and the PCR products were purified, and finally the insert size of the library was measured and quantified again using an Agilent 2100 bioanalyzer. High quality libraries were sequenced on the Illumina NovaSeq 6000 (Illumina, USA) platform to produce 150 bp paired end reads.

The raw data generated by sequencing was quality controlled using fastp (version 0.19.7) to remove splices and low quality, to obtain high quality clean data. The clean reads were then mapped to the *F. nilgerrensis* genome using Hisat2v2.0.5, featureCounts (1.5.0-p3) was used to calculate the number of reads mapped to each gene, and FPKM was calculated for each gene based on gene length. New transcripts were also predicted using StringTie (1.3.3b) [86]. The DESeq2 R package (1.20.0) was used to perform DEGs analysis between two comparative combinations at four time points, and genes with adjusted *P*-value < 0.05 (padj < 0.05) while the difference multiplier satisfied |log2(FoldChange)| >= 1 were considered as DEGs. GO and KEGG enrichment analysis of DEGs was performed using software consistent with the DNA methylation enrichment analysis.

STEM version 1.3.13 was used to analyze the temporal specificity of drought resistance gene expression by the STEM clustering method, up to 50 model profiles and all other parameters set to default values [31]. Based on five physiological traits, we performed a co-expression network using WGCNA (R/WGCNA version 1.70.3) with the filtered (FPKM > 1) 18,542 genes [87]. Parameters were set up as follows: networkType was set to signed, minModuleSize to 30, power to 17, and MEDissThres to 0.25. The networks were visualized using Cytoscape v.3.7.1. The drawing software was mainly Graphpad and ImageGP [87, 88]. The heat map was drawn with TBtools software [89].

### qRT-PCR analysis

High quality RNA was extracted from *F. nilgerrensis* leaves and reversely transcribed into cDNA and used as template for qRT-PCR. Consistent with the qRT-PCR method described in our previous study [90], *FnACTIN* was selected as an internal reference to normalize the relative expression of each gene. The primer sequences used for qRT-PCR validation are shown in Supplementary Table S7. 

## Supplementary Information

**Additional file 1:** **Table S1.** Statistics of RNA-seq reads. **Table S2.** Data description of BS-Seq reads for the four *F.nilgerrensis* samples with three replicates. **Table S3.** Whole-genome methylation of * F. nilgerrensis*. **Table S4-1.**The promoter regions DMGs and DEGs shared genes at all time points. **Table S4-2.**The genebody regions DMGs and DEGs shared genes at all time points. **Table S5.** Differentially expressed transcription factors (TF). **Table S6.** Differentially expressed transcription factors (TFs) with methylation in promoter region. **Table S7.** List of primer pairs used for qRT-PCR.

**Additional file 2:** **Figure S1. **Analysis of transcriptome results. (A) Pearsoncorrelation coefficients (R^2^) of each sample for transcriptomesequencing;(B) The quantile-quantile plot (QQ plot) quality controltest for two biological replicates of FPKM data at time point T0, with T4 ascontrol;(C) Venn diagram of differentially expressed genes (DEGs)detected by pairwise comparisons at four drought stress time points; (D)Average FPKM cluster heatmap of all differentially expressed genes at four timepoints of drought; (E) Significant expression profile changes based on ShortTime-series Expression Miner (STEM) analysis. **Figure S2. **Differentiallymethylated regions (DMRs) analysis. (A)Number of CG/CHG/CHH-DMRsdistributed in different genomic; (B) Top 10 KEGG enrichment analysis ofpromoter hypo- and hypermethylated related genes. **Figure S3. **The relationshipbetween methylation and gene expression. (A) Taking the 8th day (T8) of droughtas a representative, the comparison of expression profiles of genes withdifferent methylation levels and non-methylated genes is shown; the first group beingthe lowest and the fifth group the highest. (B) Identifies the association of promoter and gene bodymethylation with the expression of 835 genes. C1: Hypermethylation and lowexpression; C2: Hypomethylation and high expression; C3: Hypermethylation andhigh expression; C4: Hypomethylation and low expression. **Figures S4. **Phylogenetic tree (NJ-tree)analysis of the SnRK2 gene family in *F. nilgerrensis*, rice and *Arabidopsis*. Bootstrap values(%) for 1000 replicates are indicated at the nodes. I, II, III respectivelyrepresents subclass; Red squares represent *Arabidopsis*, green trianglesrepresent rice, and green circles represent *F. nilgerrensis*;The expressionheat map of SnRK2gene of the identified *F. nilgerrensis *speciesis shown. **Figure S5. **Transcriptomic and physiological traitscorrelation analysis of drought stress in *F. nilgerrensis*. (A)Dendrogram showing co-expression modules (clusters) at four time points ofdrought stress as determined by weighted correlation network analysis (WGCNA);(B) KEGG enrichment results for the top 20 of four significantly highlycorrelated modules; (C) Correlation network of highly correlated orangemodules. Cytoscape shows the top 200 genes with edge weights. The red circlesrepresent possible hub genes, and the weight is represented by the size of thenode, which reflects the number of genes related to it.

## Data Availability

Additional Supporting Information may be found online in the supporting information tab for this article: The raw data have been deposited in the NCBI Sequence Read Archive (SRA) (https://ncbi.nlm.nih.gov/subs/sra) with an accession number of PRJNA848242.

## Abbreviations

DEGs: Differentially expressed genes
DMEGs: Differentially methylated expressed genes
DMGs: Differentially methylated genes
WGCNA: Weighted gene co-expression network analysis
SOD: Superoxide dismutase
POD: Peroxidase
CAT: Catalase
ROS: Reactive oxygen species
ABA: Abscisic acid
JA: Jasmonic acid
TEs: Transposable elements
PIP: Plasma membrane intrinsic protein
MDA: Malondialdehyde
REC: Relative electrical conductivity
KEGG: Kyoto Encyclopedia of Genes and Genomes
GO: Gene Ontology
TSS: Transcription start site
TES: Transcription termination site
PYR/PYL/RCAR: PYRABACTIN RESISTANCE/PYR-LIKE/REGULATORY COMPONENTS OF ABA RECEPTORS
PP2C: Clade A type 2 C protein phosphatase
SnRK2: SNF1-related protein kinase 2
PEG: Polyethylene glycol
FBPase: Fructose 1,6-bisphosphatase
CBP: Chlorophyll-binding protein
RuBisco: Ribulose-1,5-bisphosphate carboxylase
RBOH: Respiratory burst oxidase homologs
GST: Glutathione transferase
GR: Glutathione reductase
GPX: Glutathione peroxidase
TFs: Transcription factors
DREB: Dehydration response element binding.
