# Supplementary material for: Integrated transcriptome and methylome analyses reveal the molecular regulation of drought stress in wild strawberry (Fragaria nilgerrensis)
Source: BMC Plant Biol. 2022 Dec 28;22:613. doi: 10.1186/s12870-022-04006-9 (PMC9795625; doi:10.1186/s12870-022-04006-9)
Supplement: Supplementary file 2 — Additional file 2: Figure S1. Analysis of transcriptome results. (A) Pearsoncorrelation coefficients (R2) of each sample for transcriptomesequencing;(B) The quantile-quantile plot (QQ plot) quality controltest for two biological replicates of FPKM data at time point T0, with T4 ascontrol;(C) Venn diagram of differentially expressed genes (DEGs)detected by pairwise comparisons at four drought stress time points; (D)Average FPKM cluster heatmap of all differentially expressed genes at four timepoints of drought; (E) Significant expression profile changes based on ShortTime-series Expression Miner (STEM) analysis. Figure S2. Differentiallymethylated regions (DMRs) analysis. (A)Number of CG/CHG/CHH-DMRsdistributed in different genomic; (B) Top 10 KEGG enrichment analysis ofpromoter hypo- and hypermethylated related genes. Figure S3. The relationshipbetween methylation and gene expression. (A) Taking the 8th day (T8) of droughtas a representative, the comparison of expression profiles of genes withdifferent methylation levels and non-methylated genes is shown; the first group beingthe lowest and the fifth group the highest. (B) Identifies the association of promoter and gene bodymethylation with the expression of 835 genes. C1: Hypermethylation and lowexpression; C2: Hypomethylation and high expression; C3: Hypermethylation andhigh expression; C4: Hypomethylation and low expression. Figures S4. Phylogenetic tree (NJ-tree)analysis of the SnRK2 gene family in F. nilgerrensis, rice and Arabidopsis. Bootstrap values(%) for 1000 replicates are indicated at the nodes. I, II, III respectivelyrepresents subclass; Red squares represent Arabidopsis, green trianglesrepresent rice, and green circles represent F. nilgerrensis;The expressionheat map of SnRK2gene of the identified F. nilgerrensis speciesis shown. Figure S5. Transcriptomic and physiological traitscorrelation analysis of drought stress in F. nilgerrensis. (A)Dendrogram showing co-expression modules (clusters) at four tim [file 12870_2022_4006_MOESM2_ESM.docx]

**Integrated transcriptome and methylome analyses reveal the molecular regulation of drought stress in wild strawberry (*****Fragaria nilgerrensis*)**

Qiang Cao^1#^, Lin Huang^1#^, Jiamin Li^1#^, Peng Qu^1^, Pang Tao^2^, M. James C. Crabbe^3,4,5^, Ticao Zhang^6^*, Qin Qiao^7^*

1. School of Agriculture, Yunnan University, Kunming 650091, China
2. Horticultural Research Institute, Yunnan Academy of Agricultural Sciences, Kunming 650205, China
3. Wolfson College, Oxford University, Oxford, OX26UD, UK
4. Institute of Biomedical and Environmental Science & Technology, School of Life Sciences, University of Bedfordshire, Park Square, Luton LU1 3JU, UK
5. School of Life Science, Shanxi University, Taiyuan 030006, Shanxi, China
6. College of Chinese Material Medica, Yunnan University of Chinese Medicine, Kunming 650500, China
7. College of Horticulture and Landscape, Yunnan Agricultural University, Kunming, China

^#^Q.C., L.H, J.L. contributed equally to this work.

^*^Correspondence: Q.Q. (qiaoqin@ynu.edu.cn) and T.Z. (ticaozhang@126.com)
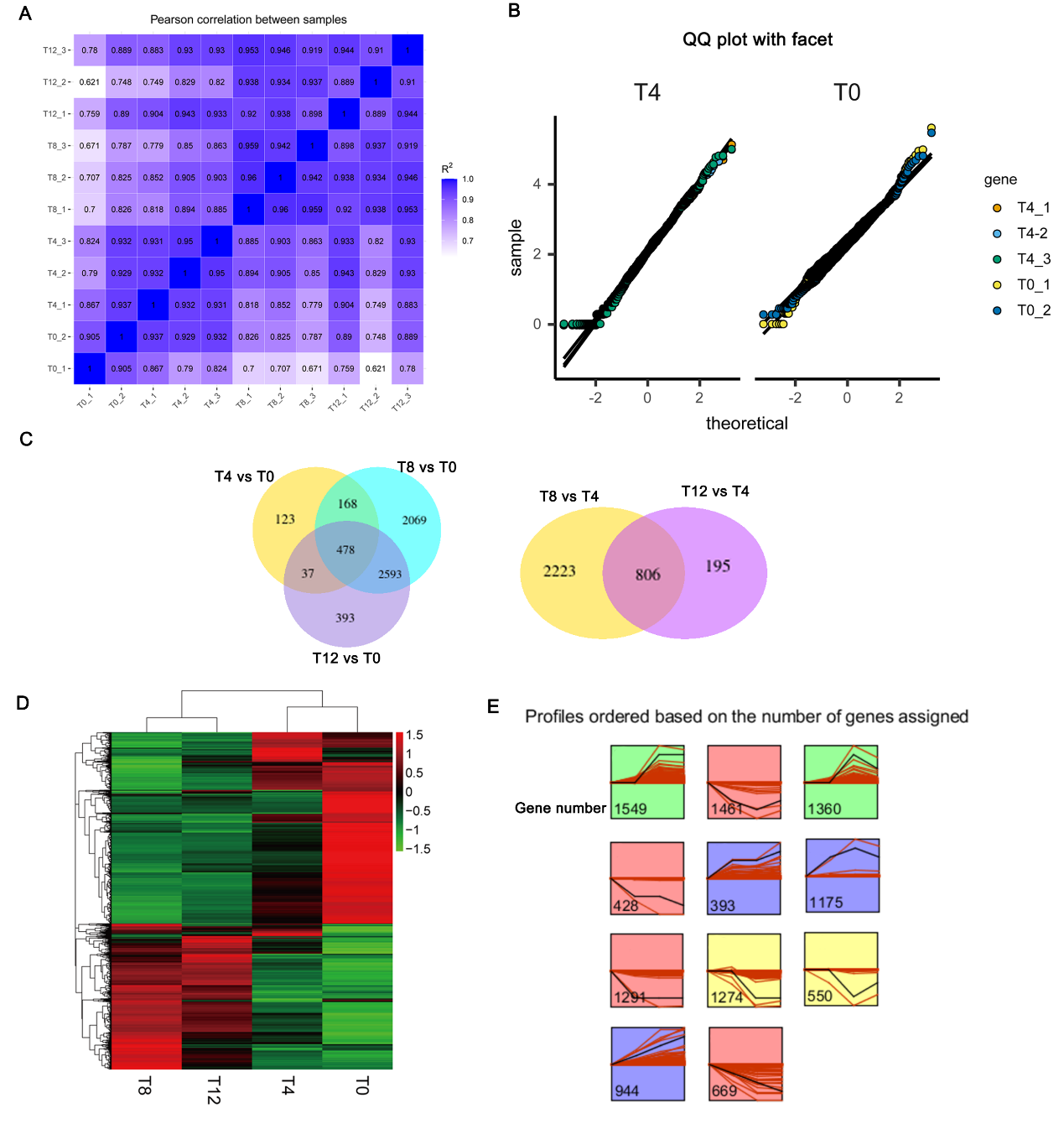


**Figure S1.** Analysis of transcriptome results. (A) Pearson correlation coefficients (R^2^) of each sample for transcriptome sequencing; (B) The quantile-quantile plot (QQ plot) quality control test for two biological replicates of FPKM data at time point T0, with T4 as control; (C) Venn diagram of differentially expressed genes (DEGs) detected by pairwise comparisons at four drought stress time points; (D) Average FPKM cluster heatmap of all differentially expressed genes at four time points of drought; (E) Significant expression profile changes based on Short Time-series Expression Miner (STEM) analysis.


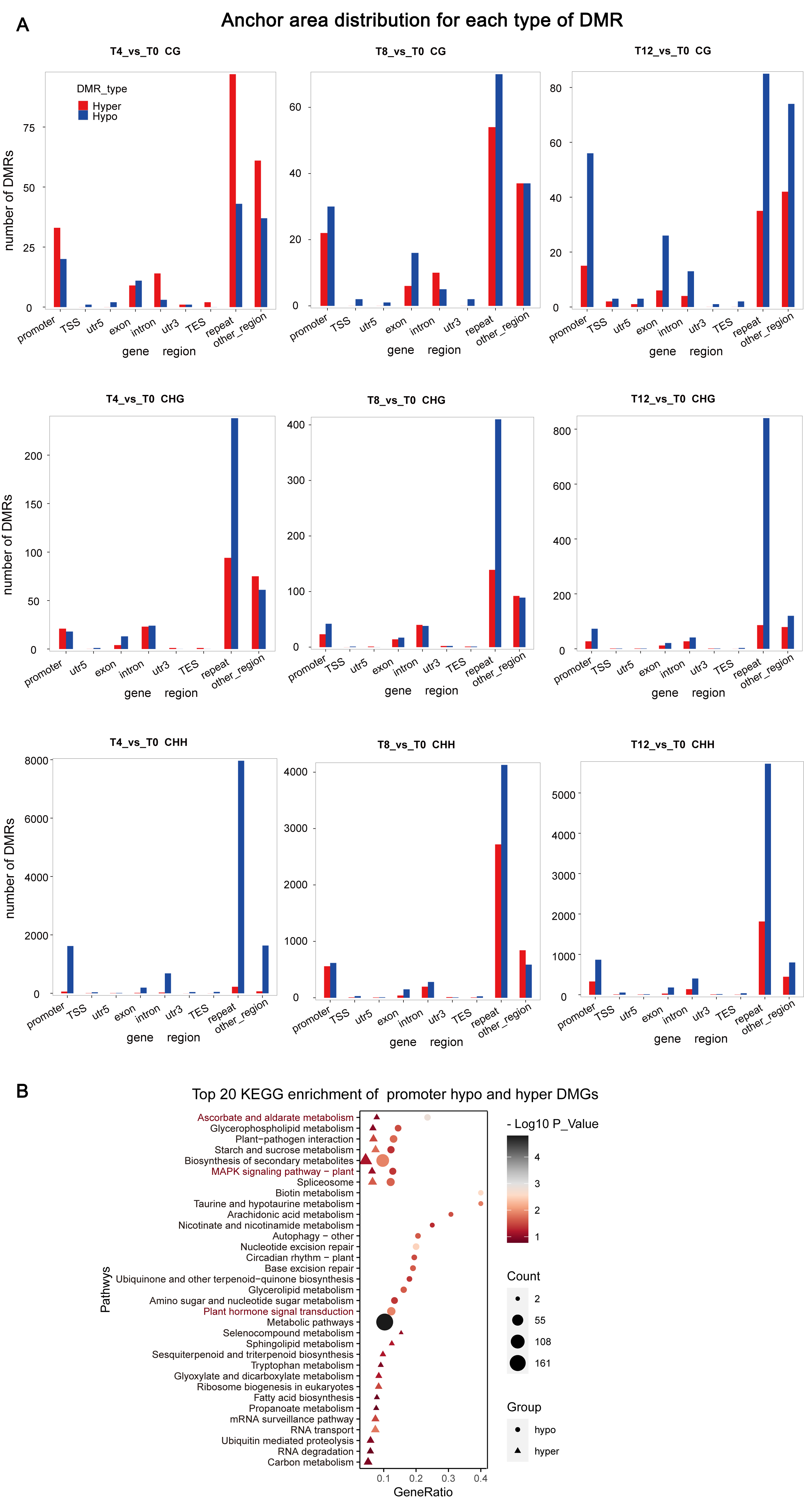


**Figure S2.** Differentially methylated regions (DMRs) analysis. (A) Number of CG/CHG/CHH-DMRs distributed in different genomic; (B) Top 10 KEGG enrichment analysis of promoter hypo- and hypermethylated related genes.


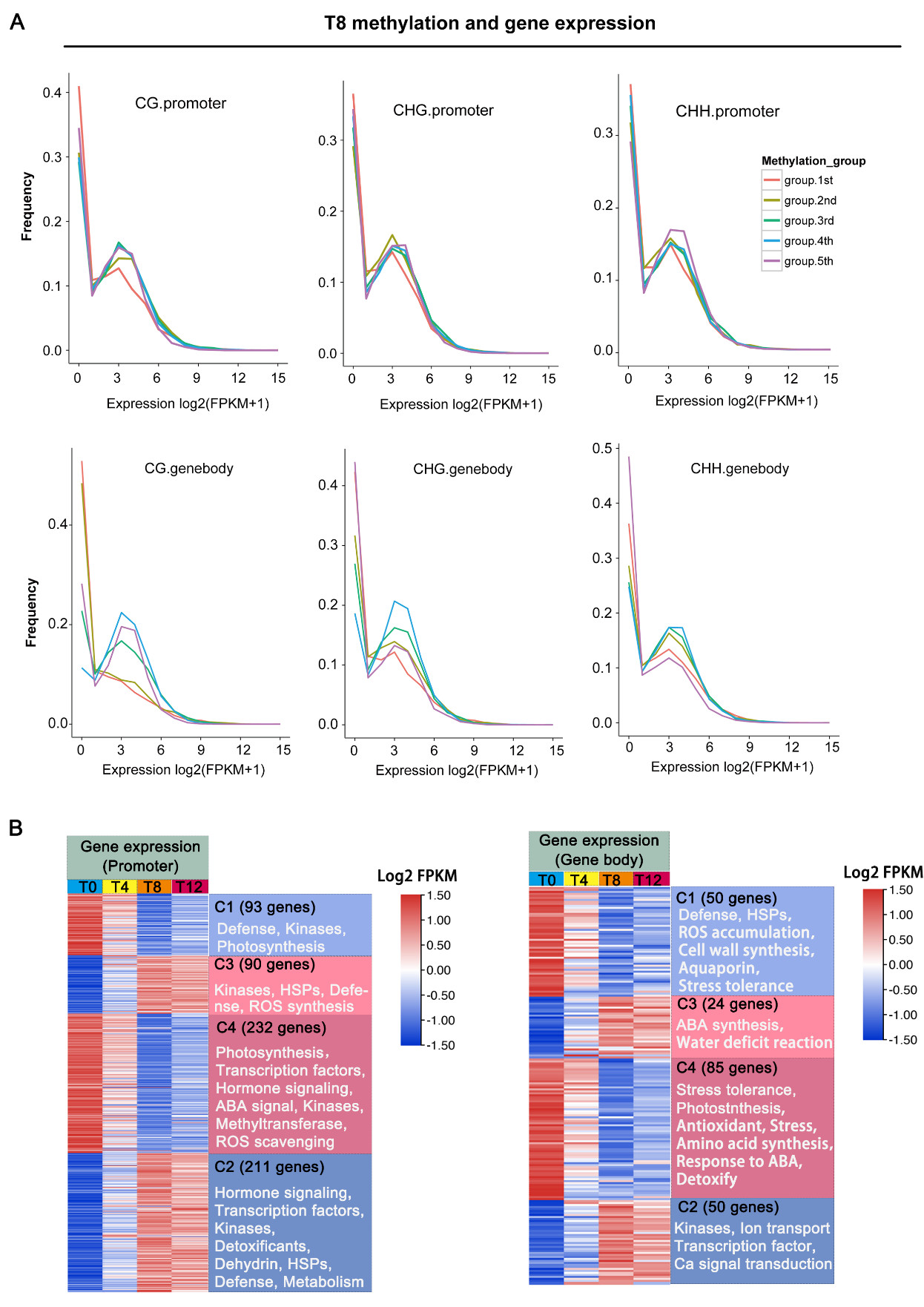


**Figure S3.** The relationship between methylation and gene expression. (A) Taking the 8th day (T8) of drought as a representative, the comparison of expression profiles of genes with different methylation levels and non-methylated genes is shown; the first group being the lowest and the fifth group the highest. (B) Identifies the association of promoter and gene body methylation with the expression of 835 genes. C1: Hypermethylation and low expression; C2: Hypomethylation and high expression; C3: Hypermethylation and high expression; C4: Hypomethylation and low expression.


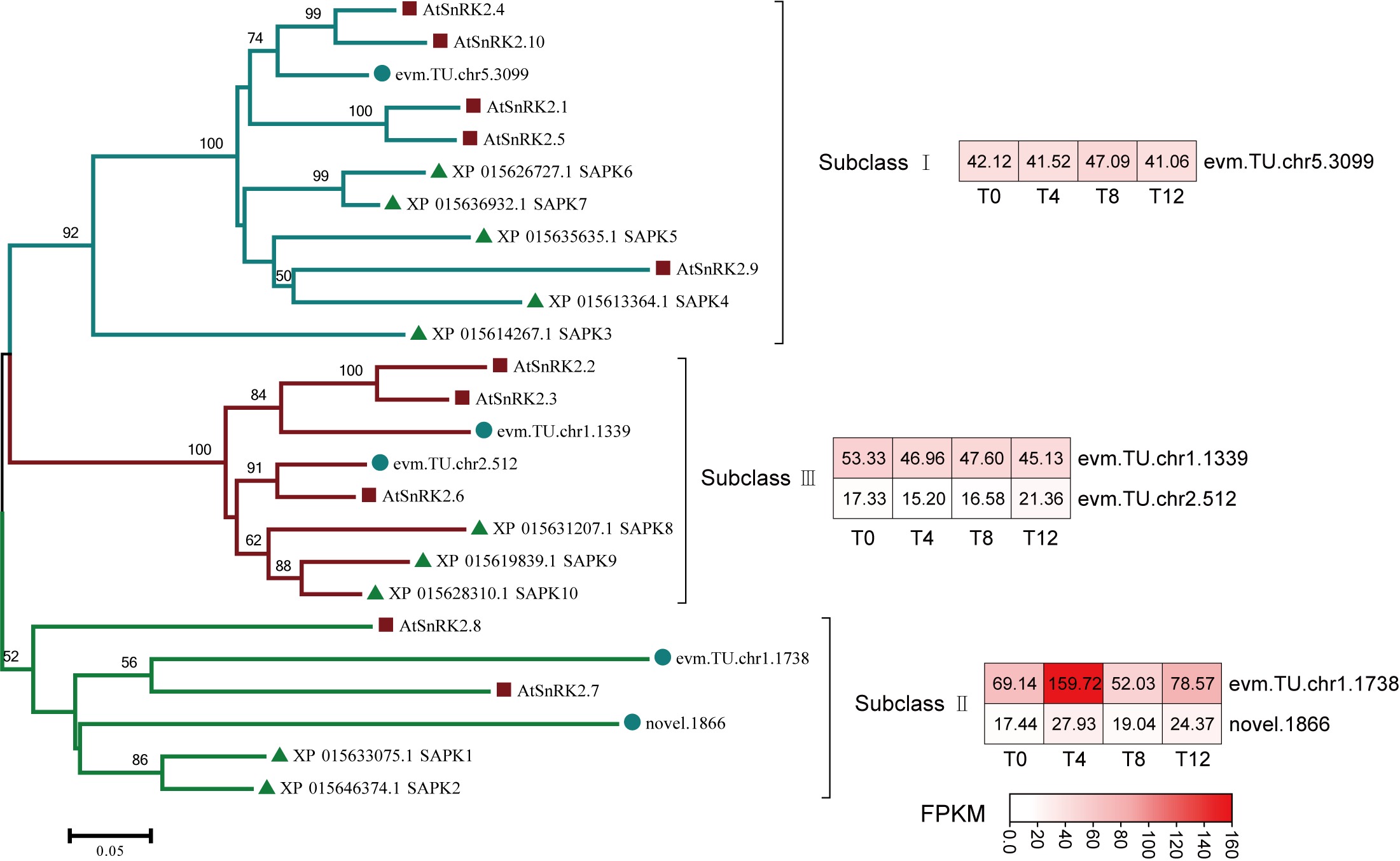


**Figures S4.** Phylogenetic tree (NJ-tree) analysis of the SnRK2 gene family in *F. nilgerrensis*, rice and *Arabidopsis*. Bootstrap values (%) for 1000 replicates are indicated at the nodes. Ⅰ, Ⅱ, Ⅲ respectively represents subclass; Red squares represent *Arabidopsis*, green triangles represent rice, and green circles represent *F. nilgerrensis*; The expression heat map of SnRK2 gene of the identified *F. nilgerrensis* species is shown.

**
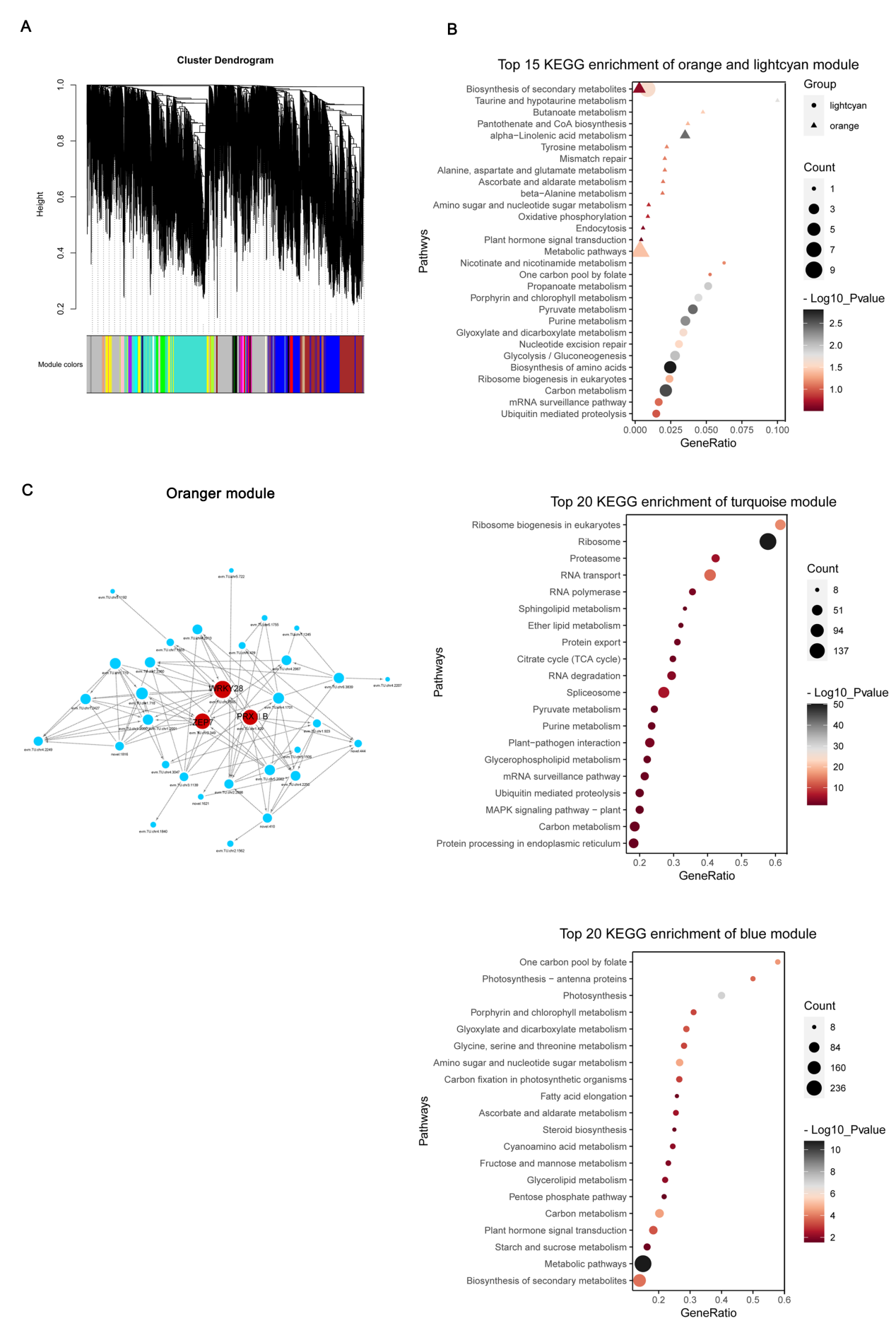
**

**Figure S5.** Transcriptomic and physiological traits correlation analysis of drought stress in *F. nilgerrensis*. (A) Dendrogram showing co-expression modules (clusters) at four time points of drought stress as determined by weighted correlation network analysis (WGCNA); (B) KEGG enrichment results for the top 20 of four significantly highly correlated modules; (C) Correlation network of highly correlated orange modules. Cytoscape shows the top 200 genes with edge weights. The red circles represent possible hub genes, and the weight is represented by the size of the node, which reflects the number of genes related to it.
